# Autocrine CXCL8-dependent invasiveness triggers modulation of actin cytoskeletal network and cell dynamics

**DOI:** 10.18632/aging.102733

**Published:** 2020-01-27

**Authors:** Andrea Antonosante, Laura Brandolini, Michele d’Angelo, Elisabetta Benedetti, Vanessa Castelli, Mattia Del Maestro, Sabino Luzzi, Antonio Giordano, Annamaria Cimini, Marcello Allegretti

**Affiliations:** 1 Department of Life, Health and Environmental Sciences, University of L’Aquila, L’Aquila, Italy; 2 Dompé Farmaceutici SpA, L’Aquila, Italy; 3 San Matteo Hospital, University of Pavia, Pavia, Italy; 4 Department of Medical Biotechnologies, University of Siena, Siena, Italy; 5 Sbarro Institute for Cancer Research and Molecular Medicine and Center for Biotechnology, Temple University, Philadelphia, PA 19122, USA

**Keywords:** glioblastoma, chemokine, CXCL8, cytoskeleton, cell migration

## Abstract

Glioblastoma (GB) is the most representative form of primary malignant brain tumour. Several studies indicated a pleiotropic role of CXCL8 in cancer due to its ability to modulate the tumour microenvironment, growth and aggressiveness of tumour cell. Previous studies indicated that CXCL8 by its receptors (CXCR1 and CXCR2) induced activation of the PI3K/p-Akt pathway, a crucial event in the regulation of cytoskeleton rearrangement and cell mobilization. Human GB primary cell culture and U-87MG cell line were used to study the effects of CXCR1 and CXCR2 blockage, by a dual allosteric antagonist, on cell migration and cytoskeletal dynamics. The data obtained point towards a specific effect of autocrine CXCL8 signalling on GB cell invasiveness by the activation of pathways involved in cell migration and cytoskeletal dynamics, such as PI3K/p-Akt/p-FAK, p-cortactin, RhoA, Cdc42, Acetylated α-tubulin and MMP2. All the data obtained support the concept that autocrine CXCL8 signalling plays a key role in the activation of an aggressive phenotype in primary glioblastoma cells and U-87MG cell line. These results provide new insights about the potential of a pharmacological approach targeting CXCR1/CXCR2 pathways to decrease migration and invasion of GB cells in the brain parenchyma, one of the principal mechanisms of recurrence.

## INTRODUCTION

Glioblastoma (GB) is the most representative form of primary malignant brain tumour. The aggressive and invasive nature of GB and the refractoriness to the standard therapy lead to a high mortality rate of GB patients with a median overall survival of about 15-16 months [1–6]. Surgical resection of the tumour mass may fail to completely remove the infiltrative disease due to the risk of functional loss thus residual malignant cells may eventually contribute to tumour recurrence. Development of specific and effective therapies requires a deep understanding of the pathways governing the invasive behaviour of gliomas. The tremendous medical need has prompted plenty of research studies on the complex biological mechanisms accounting for glioma tumour cell invasiveness leading to the identification of several orchestrated signalling pathways implicated in this phenomenon [7]. There is strong evidence that several soluble factors contribute to the gliomagenesis and particular attention has been given to a potential role of chemokines in the regulation of tumour cell invasiveness due to the specific role of this protein family in the migration of circulating immune cells [8].

Although CXCL8 was originally characterized as a leucocyte chemo-attractant, several studies have contributed to demonstrate a pleiotropic role in cancer biology due to its ability to modulate the tumour microenvironment by recruiting inflammatory leukocytes, favouring angiogenesis, modulating the growth and aggressiveness of tumour cell and specifically shielding the cancer stem cell population from the cytotoxic effect of chemotherapeutics. Atypical expression of this chemokine and its receptors is associated with a more invasive phenotype in the breast [9–11], ovarian [12, 13], pancreatic [14, 15], thyroid [16, 17] and other cancers [18–20]. The expression of CXCL8 by melanoma cells has been shown to regulate growth and metastasis in nude mice [21]. CXCL8 constitutive expression in human colon carcinoma cell lines has been linked to the metastatic potential in immunodeficient transgenic mice models, thus suggesting a role of CXCL8 in the development of distant metastases from colorectal tumours [22, 23]. Moreover, Fang et al. [24] demonstrated the paracrine role of CXCL8 secreted by TAMs (tumour-associated macrophages) in thyroid cancer progression. In addition, the Authors observed an increase of metastasis formation and the reduction of survival in male NOD/SCID mice injected with a PTC (papillary thyroid carcinoma) cell line (exactly the BCPAP cell line) following exposure to human recombinant CXCL8 (hrCXCL8).

Involvement of CXCL8 in the development and progression of glioblastoma has been extensively studied and reviewed [25–27]. Several studies have shown that CXCL8 is abundantly expressed by GB cell lines mainly as a consequence of the aberrant activation of NF-κB [26, 28–30] suggesting that CXCL8 autocrine or paracrine signalling may play a role in GB growth and invasiveness.

CXCR1 and CXCR2 are two receptors (about 76% of sequence homology) directly involved in human physiological and pathological signalling of CXCL8. These receptors are part of the seven transmembrane G-protein coupled receptors (GPCRs). CXCR1 is highly specific for CXCL8 and the binding occurs with high affinity, whereas CXCR2 can bind also other CXC chemokines. CXCL8 interaction with its cognate receptors resulting in activation of downstream pathways that can be similar but also different, therefore CXCR1 and CXCR2 can carry out the dissimilar physiological role [31].

Colon carcinoma cells show a peculiar invasive phenotype closely coupled to CXCL8/CXCR1 signalling transduction pathway, as previously demonstrated [32]. Several glioma cell lines (D54, LN229, U-87MG and U251) express only CXCR1 but not CXCR2, as already demonstrated [30]. Moreover, the same authors [30] observed the invasion reduction in glioma cell lines exposed to anti-CXCL8 or anti-CXCR1-neutralizing antibodies.

More recently, the central role of CXCL8/CXCR2 axis in stimulating therapy-dependent GB self-renewal has been demonstrated. GB self-renewal has been found associated with altered epigenetic regulation of CXCL8 that in turn induces the shift of the GB phenotype, from a differentiated state to an undifferentiated state, resulting in GB plasticity and chemotherapy resistance improvements [33].

External and internal inputs affect cytoskeletal dynamics due to the activation of various signal transduction cascades. The arrangement and combination of these signal transduction events depict the underlying mechanisms that regulate cytoskeleton networks functions. These mechanisms require the activation of multiple actin and microtubules modifiers including kinases, GTPases and other effectors and regulators [34, 35]. Cytoskeletal dynamics, which is required to promote the local invasion of glioblastoma, seems to be dependent upon the different proteins interactions with cytoskeletal components [7, 36, 37]. Previous literature data support the concept that CXCR1 induced activation of the PI3K/p-Akt pathway, which is a crucial event in the regulation of cytoskeleton rearrangement and cell mobilization in cancer [38, 39].

Starting from these evidences, in this work we first examined the expression of CXCR1/CXCR2 and secreted CXCL8 in human glioblastoma U-87MG cell line and primary cell cultures from post-surgical specimens of glioblastoma patients. Then, we studied the role of CXCL8 signalling in sustaining the invasiveness and aggressiveness of GB using a dual CXCR1/CXCR2 allosteric inhibitor, which blocks the activation of both receptor subtypes by binding a highly conserved allosteric site.

The data obtained point towards a specific effect of autocrine CXCL8 signalling on GB cell invasive behaviour mediated by the activation of the molecular mechanisms involved in cell migration and cytoskeletal dynamics, such as PI3K/p-Akt/p-FAK, p-cortactin, RhoA, Cdc42, Acetylated α-tubulin and MMP2.

## RESULTS

### The GB cellular models show different levels of CXCL8 and CXCR1/CXCR2 associated with autocrine CXCL8 signalling

**Figure 1:**
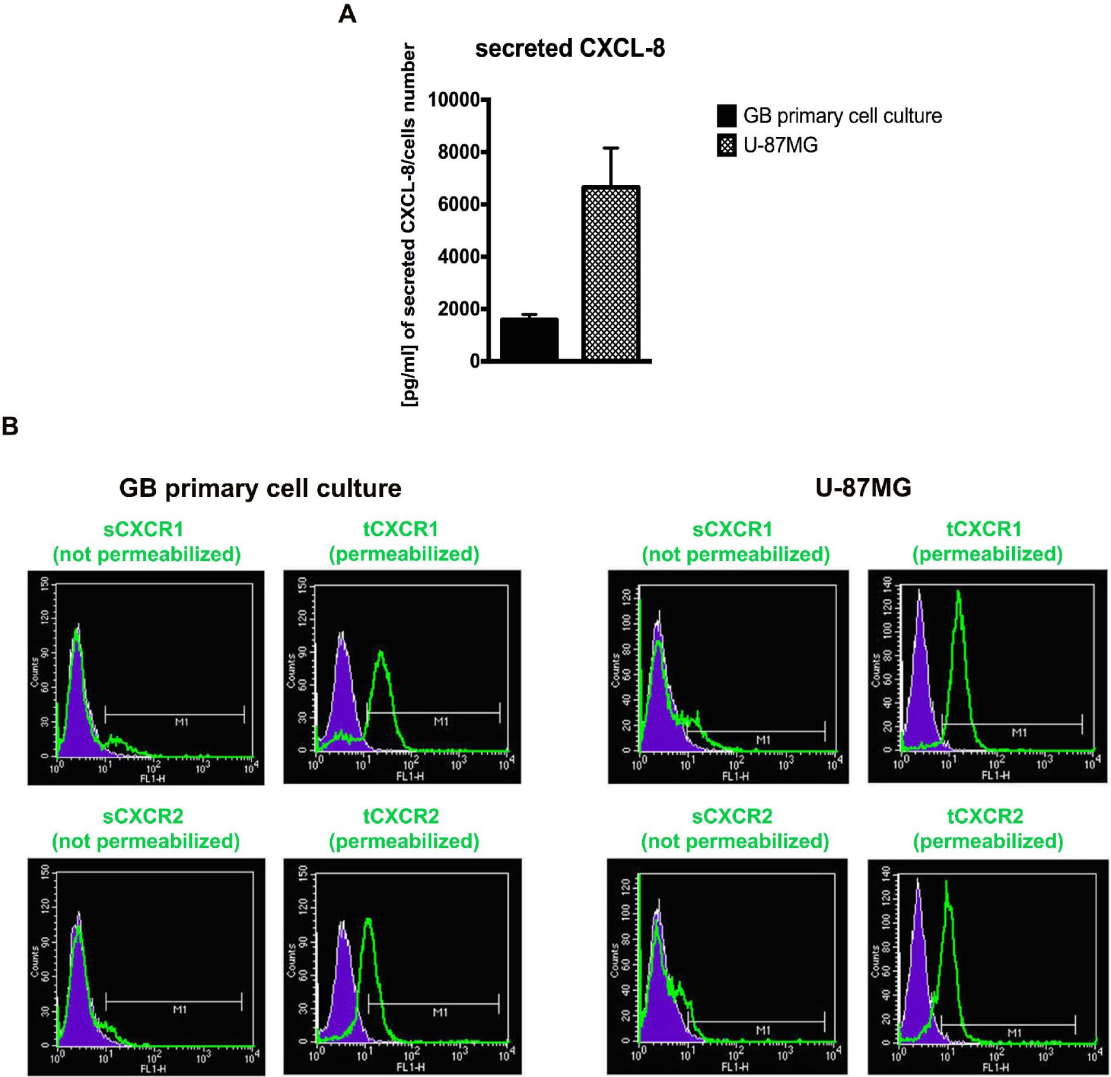
**The GB cellular models show different levels of CXCL8 and CXCR1/2.** ELISA assay was used to quantify the amount of CXCL8 secreted in the supernatant media from GB primary cell culture and U-87MG cells (**A**). Data are means ± SEM of three different biological replicates (n=3). (**B**) Representative cytofluorimetric analysis for CXCR1 and CXCR2 protein levels in GB primary cell culture and U-87MG cell line. Cytofluorimetric profile images are representative one. Cytofluorimetric analysis were performed in permeabilized or not permeabilized cells. tCXCR1/2: total protein levels in permeabilized cellular samples; sCXCR1/2: surface protein levels in not permeabilized cellular samples.

In the first series of experiments, GB primary cell cultures from patient specimens were characterized to confirm the presence of typical glioma markers, such as GFAP and SOX2 (Supplementary Figure 1). Then, CXCR1/CXCR2 expression and extracellular CXCL8 levels (in supernatant from U-87MG and GB primary cell cultures) were analysed to evaluate the autocrine CXCL8 mediated signalling in GB. In Figure 1A ELISA detection of secreted CXCL8 in culture media from GB primary cell culture and U-87MG cells is reported together with the cytofluorimetric analysis for CXCR1 and CXCR2 content in both cellular models (Figure 1B). The presence of the receptors was investigated in permeabilized and not permeabilized cells showing that the surface receptors are quickly internalized in a CXCL8-dependent manner [31, 40, 41]. The CXCR1/CXCR2 trafficking between membrane surface and cytoplasm is strictly regulated as well as their G-protein signalling. Both receptors are promptly desensitized to avoid constitutive signalling activation. CXCR1 internalizes slower than CXCR2, also when the ligand concentration is low. At the same time CXCR1 is recycled on the membrane faster than CXCR2 [31, 41]. In coherence with the described mechanism, our *in vitro* data show high CXCR1/ CXCR2 overall levels (in permeabilized cells) as compared to low CXCR1/CXCR2 surface levels (in not permeabilized cells), due to their peculiar membrane turnover and cellular trafficking. This evidence is consistent with the high CXCL8 levels detected in the medium and in line with the hypothesis that an autocrine CXCL8-induced signalling, involving both CXCR1 and CXCR2, is activated in GB.

### CXCR1/CXCR2 allosteric inhibition elicits suppression of the invasiveness and migration without cytotoxic effect in GB cells

In the second set of experiments, the dose-dependent effect of DF2755A, a potent and selective dual CXCR1/CXCR2 non competitive allosteric inhibitor [42], was assayed in 0.1-5 μM concentration range on cell viability (Supplementary Figure 2). No evident cytotoxic effects were observed at any concentration used; on this basis, the 0.1 μM concentration for 24 hours was chosen as the experimental condition for the subsequent experiments.

**Figure 2:**
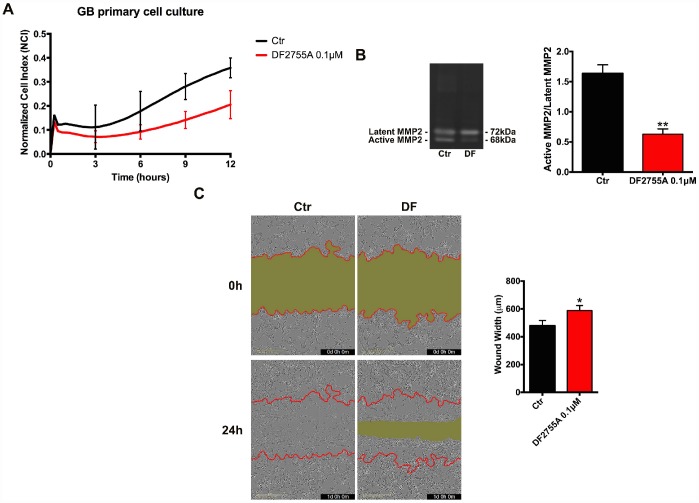
**Cell chemotaxis assay in GB primary cell culture under DF2755A treatment.** (**A**) Normalized cell index after 24 hours of treatment, the cell migration was followed for 12 hours. The supernatants of chemotaxis assay were collected to perform gelatin zymography. In (**B**) a representative gelatin zymography and relative densitometry analysis expressed as relative units of active MMP2/latent MMP2 ratio. (**C**) Representative images of wound closure at 0 hours (top) and 24 hours (bottom), the red lines represent the edges of the starting scratch, while the green areas represent the wound closure. The wound analysis was represented as wound width (μm) after 24 hours of migration. Data are means ± SEM of three different biological replicates (n=3). Statistical analysis was performed by the unpaired Student's t-test (with Welch’s correction). *, p< 0.05; **, p< 0.01, Ctr vs DF2755A were considered statistically significant. Ctr: Control, DF: DF2755A 0.1 μM. Scale bar = 400 μm.

**Figure 3:**
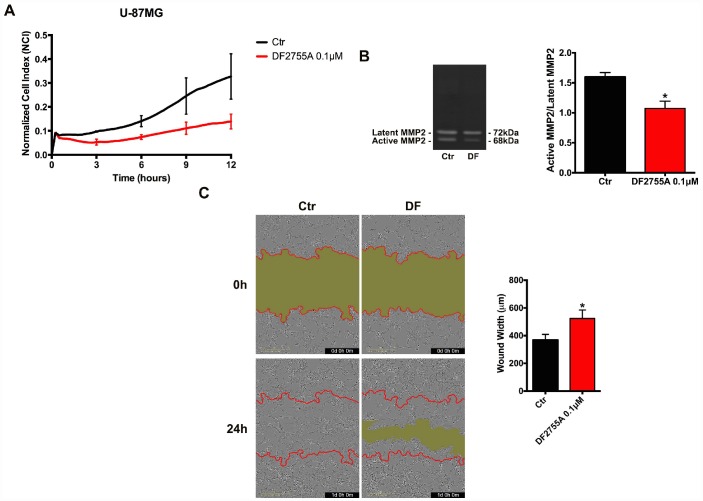
**Cell chemotaxis assay in U-87MG cells under DF2755A treatment.** (**A**) Normalized cell index after 24 hours of treatment, the cell migration was followed for 12 hours. The supernatants of chemotaxis assay were collected to perform gelatin zymography. In (**B**) a representative gelatin zymography and relative densitometry analysis expressed as relative units of active MMP2/latent MMP2 ratio. (**C**) Representative images of wound closure at 0 hours (top) and 24 hours (bottom), the red lines represent the edges of the starting scratch, while the green areas represent the wound closure. The wound analysis was represented as wound width (μm) after 24 hours of migration. Data are means ± SEM of three different biological replicates (n=3). Statistical analysis was performed by the unpaired Student's t-test (with Welch’s correction). *, p< 0.05; **, p< 0.01, Ctr vs DF2755A were considered statistically significant. Ctr: Control, DF: DF2755A 0.1 μM. Scale bar = 400 μm.

In Figures 2 and 3, the results of CXCL8-induced cell chemotaxis and zymography assays are reported for both cellular models. DF2755A treatment decreased the Normalized Cell Index (NCI) related to cell chemotaxis (Figures 2A and 3A), and significantly reduced the migration slope (about 45% in GB primary cell cultures and 60% in U-87MG cells) compared to untreated cells. The slope measures how NCI changes over time and is used to determine the rates of chemotaxis events. In Figures 2B and 3B the MMP2 activity, analysed by gelatin zymography assay, is reported. CXCL8 signalling inhibition by DF2755A administration induced, in both cellular models, the reduction of MMP2 activity expressed as active MMP2/latent MMP2 ratio. A significant decrease in the ratio was observed in DF2755A treated cells compared to untreated cells. In the same panel live imaging wound analysis of control and treated glioblastoma cells are shown. It is possible to observe that in the presence of DF2755A cell migration leading to wound closure was significantly delayed (Figures 2C and 3C). Wound width, measured by Incucyte analysis software and expressed in μm was reduced in untreated *vs* treated cells.

### DF2755A modulates the activity of the protein involved in cell motility and NF-κB p65 nuclear translocation

To evaluate a potential role of autocrine CXCL8 signalling in the modulation of GB cells aggressiveness, the effect of CXCL8 signalling blockage on the activation of key pathways related to invasiveness and invadopodia formation was investigated in unstimulated GB cell line and primary culture.

**Figure 4:**
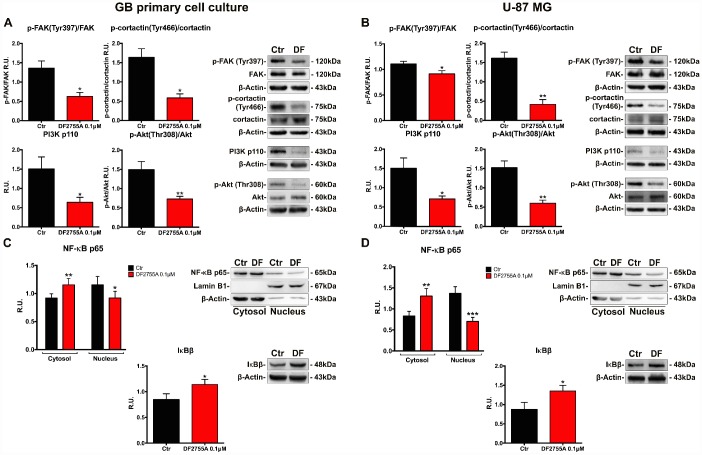
**DF2755A modulates the activity of the protein involved in cell motility and NF-κB p65 nuclear translocation.** Representative western blotting and relative densitometry analysis for p-FAK (Tyr397)/Fak, p-cortactin (Tyr466)/cortactin, PI3K, p-Akt (Thr308)/Akt in GB primary cell culture (**A**) and U-87MG cells (**B**) untreated and treated with DF2755A. Western blotting and relative densitometry analysis for cytoplasmic (normalized on the β-Actin) and nuclear (normalized on Lamin B1) NF-κB p65 and its cytoplasmic inhibitor IκBβ in GB primary cell culture (**C**) and U-87MG cells (**D**) untreated and treated with DF2755A. Data are mean ± SEM of three different biological replicates (n=3). Statistical analysis was performed by the unpaired Student's t-test (with Welch’s correction). *, p< 0.05; **, p< 0.01; ***, p< 0.001, Ctr vs DF2755A were considered statistically significant. Ctr: Control, DF: DF2755A 0.1 μM. R.U.: relative units.

Proteins involved in cytoskeletal dynamics and migration, such as the active form of focal adhesion kinase (p-FAK) and p-cortactin, were studied by western blotting in GB primary cell culture (Figure 4A) and U-87MG cell line (Figure 4B). In both cell types, DF2755A was able to decrease p-FAK, p-cortactin, PI3K/p-Akt protein levels in coherence with the effect observed in chemotaxis and migration assays. PI3K/Akt signalling activation induces several signal transduction events leading to cellular migration, cell invasion and focal adhesion formation. After CXCL8 binding, CXCR1 and CXCR2 receptors can activate the signal cascade events linked to PI3K/Akt signalling pathway and FAK phosphorylation [38, 39, 43]. DF2755A significantly decreased PI3K protein levels and p-Akt/Akt ratio in both GB *in vitro* models. In this regard, since NF-κB p65 is a downstream target of PI3K/Akt and is one of the effectors in the CXCL8 signalling pathway involved in cell invasion [26, 29, 30, 44]; it was investigated in GB primary cell culture (Figure 4C) and U-87MG cells (Figure 4D). The western blotting analysis was performed on cytosolic and nuclear protein extracts obtained by subcellular fractionation. In agreement with previous results related to PI3K/p-Akt protein levels, cytosolic NF-κB p65 protein levels were higher in treated cells compared to untreated cells in both GB cellular models used. The antagonist effect probably enhanced the NF-κB p65 cytosolic accumulation and its inactivation through interaction with its cytoplasmic inhibitor IκBβ, thus resulting in reduced NF-κB p65 nuclear translocation. DF2755A treatment significantly down regulated nuclear NF-κB p65 protein levels (the effect is more pronounced in U-87MG cells than GB primary cell culture), while increasing the protein levels of its cytoplasmic inhibitor IκBβ.

### Cytoskeletal and microtubules dynamics are adversely affected by DF2755A in GB cells

**Figure 5:**
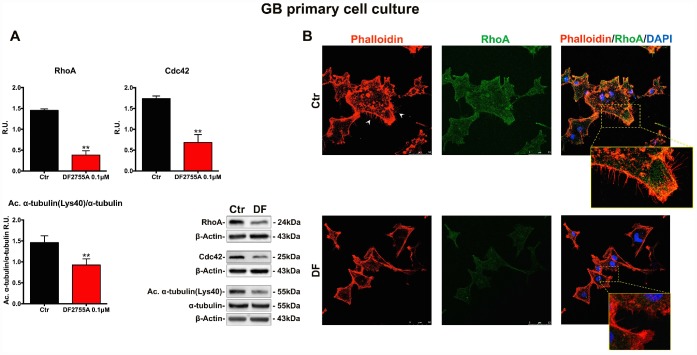
**Cytoskeletal and microtubules dynamics are adversely affected by DF2755A in GB primary cell culture.** (**A**) Representative western blotting and relative densitometry analysis for RhoA, Cdc42, Acetylated α-tubulin (Lys40)/α-tubulin. Data are mean ± SEM of three different biological replicates (n=3). Statistical analysis was performed by the unpaired Student's t-test (with Welch’s correction). **, p< 0.01, Ctr vs DF2755A were considered statistically significant. Ctr: Control, DF: DF2755A 0.1 μM. R.U.: relative units. (**B**) Immunolocalization of RhoA and microfilament decoration by phalloidin-staining in control and DF2755A treated GB primary cell cultures. White arrowheads indicate filopodia, their retraction is clear in magnified images of DF2755A treated cells. Ctr: Control, DF: DF2755A 0.1 μM. Scale bar = 50 μm.

**Figure 6:**
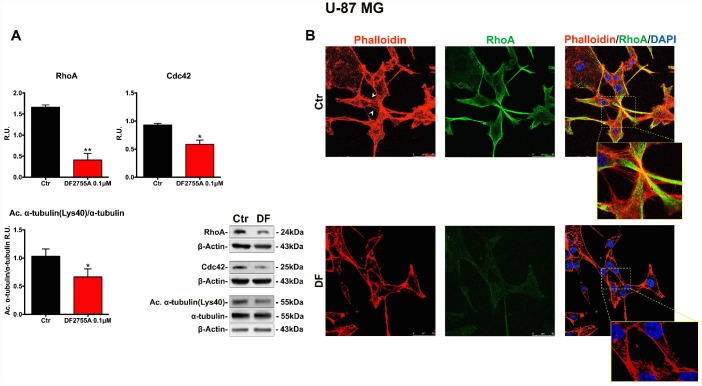
**Cytoskeletal and microtubules dynamics are adversely affected by DF2755A in U-87MG cells.** (**A**) Representative western blotting and relative densitometric analysis for RhoA, Cdc42, Acetylated α-tubulin (Lys40)/α-tubulin. Data are mean ± SEM of three different biological replicates (n=3). Statistical analysis was performed by the unpaired Student's t-test (with Welch’s correction). *, p< 0.05; **, p< 0.01, Ctr vs DF2755A were considered statistically significant. Ctr: Control, DF: DF2755A 0.1 μM. R.U.: relative units. (**B**) Immunolocalization of RhoA and microfilament decoration by phalloidin-staining in control and DF2755A treated U-87MG cell line. White arrowheads indicate filopodia, their retraction is clear in magnified images of DF2755A treated cells. Ctr: Control, DF: DF2755A 0.1 μM. Scale bar = 50 μm.

In Figures 5A and 6A, the western blotting analysis for the members of the Rho family, such as RhoA and Cdc42, in GB primary cell culture and U-87MG cells are reported, respectively. RhoA and Cdc42 protein levels were significantly decreased by DF2755A treatment. In the same Figures, the levels of acetylated α-tubulin were evaluated by western blotting analysis, being the acetylation at lysine 40 a key factor in cytoskeleton stabilization, cellular adhesion and motility. DF2755A treatment induced a significant decrease in lysine acetylation levels in both cellular models leading to a significant reduction of the acetylated α-tubulin/α-tubulin ratio, as reported in Figure 5A and 6A. To confirm CXCL8-dependent modulation of cytoskeletal dynamics, the effect of DF2755A on microfilaments localization was assessed by immunofluorescence analysis for actin (by phalloidin-staining) and RhoA using confocal laser microscopy (Figures 5B and 6B). GB primary cell culture and U-87MG cells showed characteristic filopodia, also called microspikes (indicated by white arrowheads), while allosteric inhibition of CXCL8 receptors induces a pronounced retraction of these cytoskeletal structures in both cellular models. In this regard, a strong reduction in RhoA fluorescence intensity was observed upon DF2755A treatment compared to the control cells, in agreement with the western blotting findings.

### YAP/TAZ nuclear translocation is connected with CXCL8-CXCR1/2 axis in GB cells

**Figure 7:**
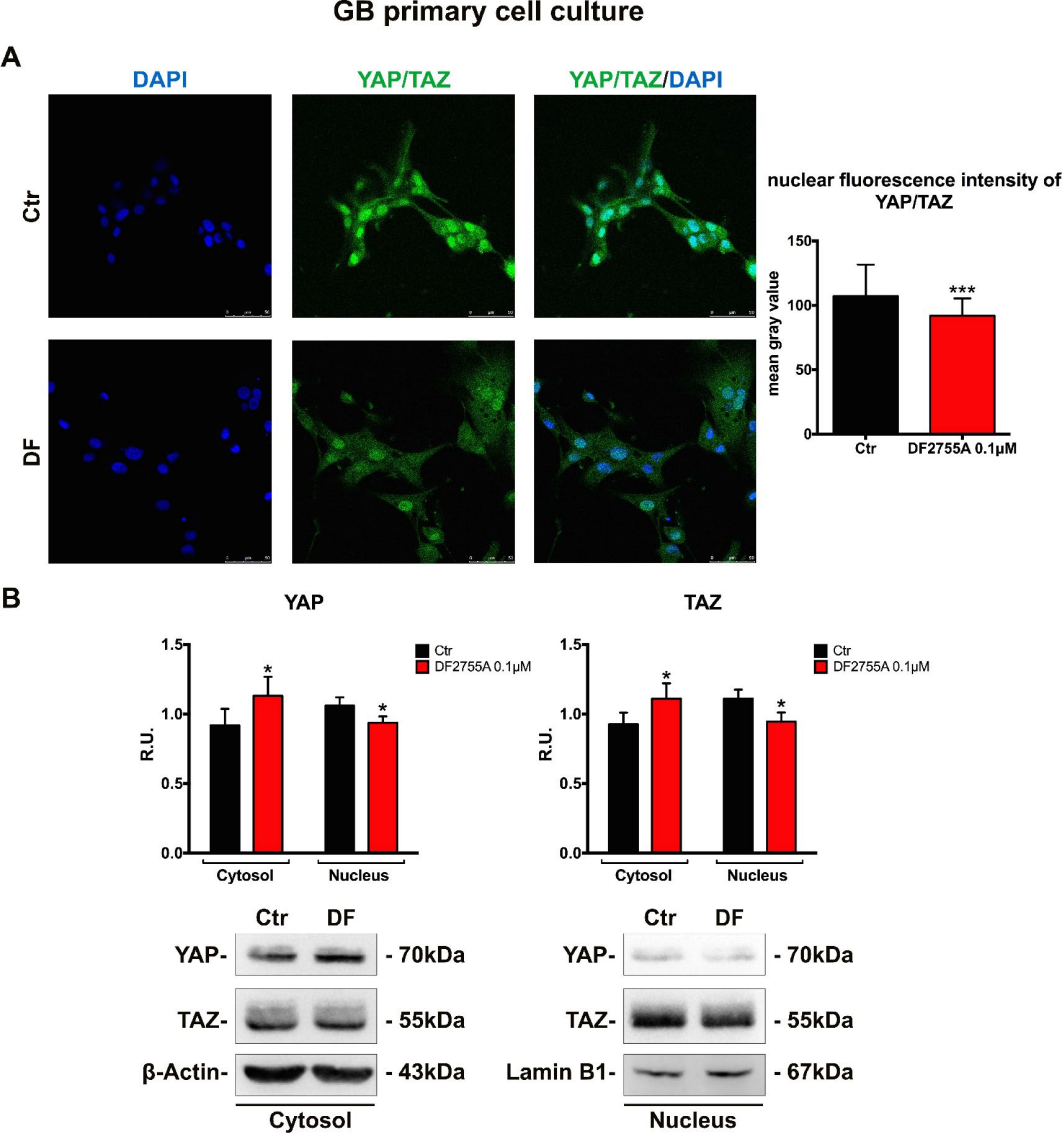
**YAP/TAZ nuclear translocation is connected with CXCL8-CXCR1/CXCR2 axis in GB primary cell culture.** (**A**) Immunolocalization of YAP/TAZ in control and treated GB primary cell culture. Ctr: Control, DF: DF2755A 0.1 μM. The nuclear fluorescence intensity quantification (indicated as mean gray value) is also reported (range of cells analysed: 117-142 for GB primary cell culture). Data are mean ±SEM of three different biological replicates (n=3). Statistical analysis was performed by the unpaired Student's t-test (with Welch’s correction). ***, p< 0.001, Ctr vs DF2755A were considered statistically significant. Scale bar = 50 μm. In (**B**) representative western blotting and relative densitometry analysis for cytoplasmic (normalized on β-Actin) and nuclear (normalized on Lamin B1) YAP and TAZ. Data are mean ±SEM of three different biological replicates (n=3). Statistical analysis was performed by the unpaired Student's t-test (with Welch’s correction). *, p< 0.05, Ctr vs DF2755A were considered statistically significant. Ctr: Control, DF: DF2755A 0.1 μM. R.U.: relative units.

**Figure 8:**
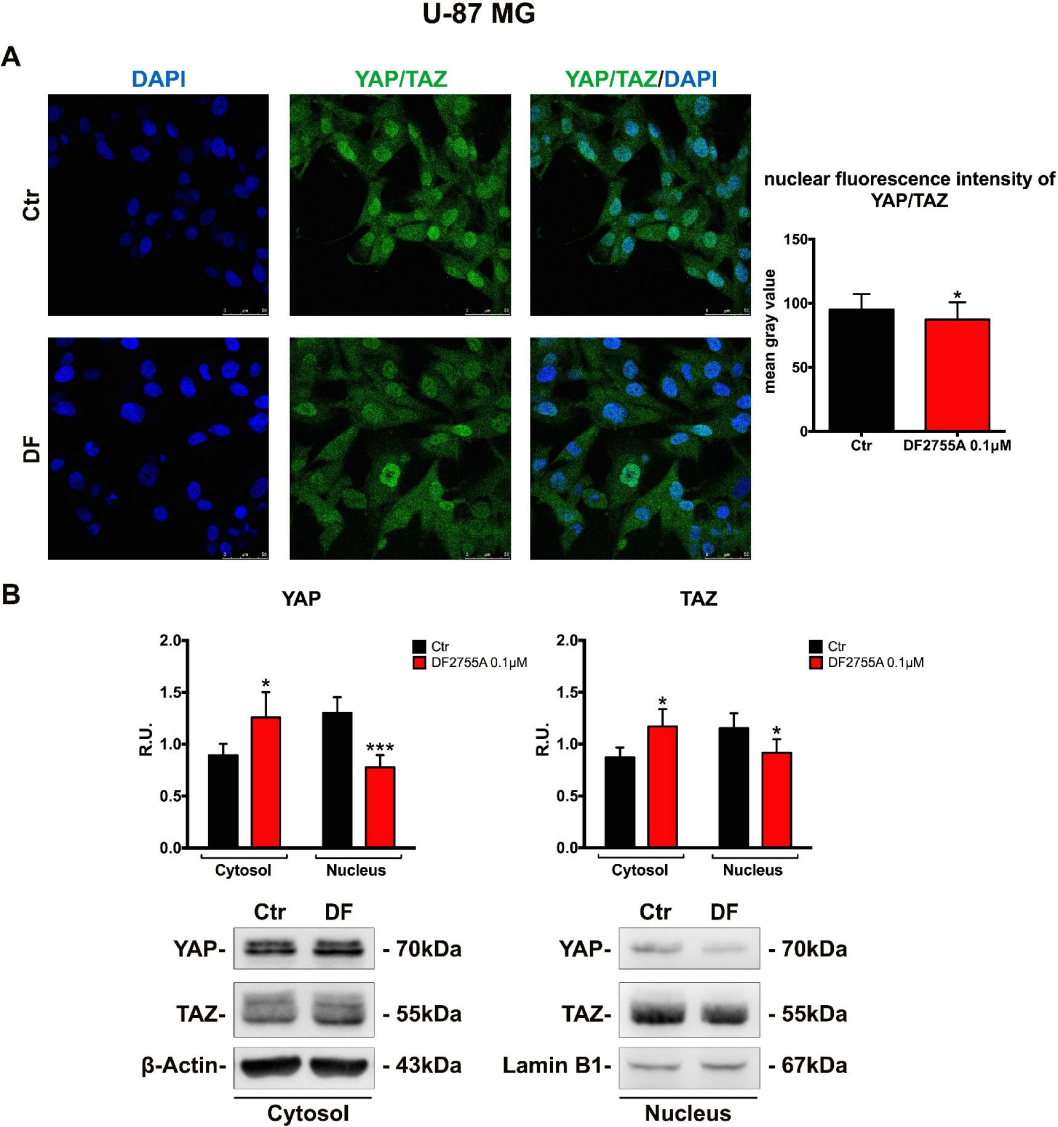
**YAP/TAZ nuclear translocation is connected with CXCL8-CXCR1/CXCR2 axis in U-87MG cell line.** (**A**) Immunolocalization of YAP/TAZ in control and treated U-87MG cells. Ctr: Control, DF: DF2755A 0.1 μM. The nuclear fluorescence intensity quantification (indicated as mean gray value) is also reported (range of cells analysed: 167-181 for U-87MG cells). Data are mean ±SEM of three different biological replicates (n=3). Statistical analysis was performed by the unpaired Student's t-test (with Welch’s correction). *, p< 0.05, Ctr vs DF2755A were considered as statistically significant. Scale bar = 50 μm. In (**B**) representative western blotting and relative densitometry analysis for cytoplasmic (normalized on β-Actin) and nuclear (normalized on Lamin B1) YAP and TAZ. Data are mean ±SEM of three different biological replicates (n=3). Statistical analysis was performed by the unpaired Student's t-test (with Welch’s correction). *, p< 0.05, ***p< 0.001, Ctr vs DF2755A were considered statistically significant. Ctr: Control, DF: DF2755A 0.1 μM. R.U.: relative units.

Finally, in order to verify if the treatment with a CXCR1/CXCR2 allosteric inhibitor may modulate the cellular motility pathway triggered by nuclear localization of the transcription factors YAP and TAZ (inhibitors of the Hippo pathway controlling cell proliferation, cell migration and organs dimension, generally deregulated in cancer); the localization of these transcription factors (detected using the same antibody due to the very high homology) was investigated in the two cellular models. In Figure 7A and 8A, the immunolocalization of YAP/TAZ in GB primary cell culture and U-87MG cells are shown, respectively. It is possible to observe that in control cells YAP/TAZ show strong nuclear staining, while upon treatment, the nuclear localization is reduced, as also indicated by the fluorescent intensity quantification. To support these results, cytosolic and nuclear protein levels of YAP/TAZ were assayed. In GB primary cell culture (Figure 7B), where the treatment with DF2755A affected the YAP/TAZ cytosol-nuclear trafficking, we observed an increase of YAP and TAZ cytosolic levels associated with a reduction of their nuclear levels. The same situation was observed in U-87MG cells (Figure 8B), except for YAP nuclear levels in treated cells, where the decrease was more pronounced than in treated GB primary cell culture.

## DISCUSSION

CXCL8 exerts pleiotropic effects in cancer being involved in tumour cells proliferation, invasiveness, angiogenesis and inflammation. Human cancers associated with high metastatic potential show constitutive expression of CXCL8, which enhances the development of metastases from primary tumours [8]. Previous literature points to a specific expression of CXCR1 and lack of CXCR2 expression in GB cell lines [30], although we observed that glioblastoma cells also express CXCR2. The results obtained by us and others may be affected by the type of antibody and experimental protocol used for flow cytometry. However, we report herein that both U-87MG and GB primary cell culture express both receptors and produce considerable levels of CXCL8 secreted in the culture media.

Interestingly, relatively low levels of CXCR1 and very low levels of CXCR2 were found on the cell surface, whereas in the intracellular compartment their detected levels are plentiful. Being the receptor internalization process activated by CXCL8 (as extensively demonstrated in transfected HEK 293 and human neutrophil cells [40, 41]), this condition combined with the abundant levels of secreted CXCL8 suggest a constitutive autocrine activation of the CXCR1/CXCR2 pathway. In consideration of the specific CXCL8-dependent membrane turnover of the two receptors, we assessed, through flow cytometry analysis, both surface and total levels of the receptors. Data obtained showing that, despite the low levels on the cell membrane, both CXCR1 and CXCR2 pathways may be active in our cellular models.

Both U-87MG and GB primary cultures were found responsive to CXCL8 in cell chemotaxis and migration assay, thus confirming the functionality of the CXCL8-CXCR1/CXCR2 axis and its potential role in modulating tumour invasiveness. The blockage of CXCL8 pathway by a known selective non-competitive allosteric CXCR1/CXCR2 inhibitor, DF2755A [42], reduces CXCL8 induced migration.

Cancer cell migration is a complicated process that needs cytoplasmic membrane protrusions, also called lamellipodia and filopodia, which are consolidated at the leading edge by new adhesions with extracellular matrix (ECM). This situation allows stress fibres contraction, which effect is reflected in the cell cortex. Later at the tailing edge, the loss of old adhesions and the subsequent cell tail retraction occurs. Cell invasiveness is strictly dependent on invadopodia, membrane extroflexions composed of cytoskeletal protein scaffoldings, which are deeply projected in the ECM [34, 35, 45]. Focal adhesions are essential elements for the cell motility process, promoting cell adherence to the extracellular matrix *via* specific integrins (members of the ECM receptor family). Modifications of these adherences between cells and ECM affect cells adhesion and migration through surrounding tissues. Focal adhesion kinase (FAK), a protein tyrosine kinase associated with the focal adhesion complex, has a crucial role in integrin- mediated cell adhesion and cell spreading in cancer invasion [46, 47].

In tumours, FAK regulates cell migration and invasion through different pathways by inducing the dynamic regulation of focal adhesion and peripheral actin structures. Autophosphorylation at tyrosine 397 is the main regulation event that triggers actin modulations involved in cell motility [47–49]. This phosphorylation is due to integrin-ECM binding and other events associated with CXCL8 binding to CXCR1/CXCR2 receptors [50]. Tyrosine 397 phosphorylation allows interactions between FAK and proteins regulating cancer cell migration and invasion, such as Src, cortactin [51] and PI3K [52]. Glioma invasiveness is ensured by the matrix metalloproteinases (MMP)-mediated matrix degradation. MMP2 and MMP9 belonging to the gelatinase subfamily are abundantly expressed and directly related to the degree of glioblastoma malignancy. Moreover, MMP2 expression and secretion are FAK-dependent mechanisms, as already demonstrated in glioma cell lines [53, 54].

FAK-dependent activation of Src can stimulate several signal transduction pathways such as PI3K-Akt, RAF/JNK, and Rho/Rac/FAK [38, 55, 56] and the activation of these signal cascades modulates cell motility and survival. FAK activation is typically upstream of Akt that plays a central role in governing cellular motility. It has been reported that, under specific circumstances, extracellular environment components stimulate cancer cell adhesion via Akt-dependent FAK activation [57]. During the cell migration process, the leading edge of the cell is involved in peripheral adhesions, whereas the back end of the cell is implicated into focal adhesions. Several studies have contributed to elucidate the implication of RhoA in the regulation of contraction and retraction forces required for cell migration [58].

External and internal inputs affect cytoskeletal dynamics due to the activation of various signal transduction cascades. The arrangement and combination of these signal transduction events depict the underlying mechanisms that regulate cytoskeleton networks functions. Cortactin has emerged as a crucial molecular scaffold that mediates the assembly and organization of actin cytoskeletal networks leading to various aspects of cell dynamics. In glioblastoma, cytoskeletal dynamics, which is required for cellular motility and invasiveness, seems to be dependent upon cortactin interaction with cytoskeletal components. The characterization and cellular functions of cortactin in actin dynamics have been extensively reviewed [59]. Cortactin is abundantly localized in invadopodia structures of invasive cancer cells due to its role in the regulation of enzymes involved in ECM degradation, such as MMPs [60]. Since its first identification as a substrate of v-Src, various kinases have also been reported to act on cortactin, mainly on tyrosine residues. Phosphorylation occurs at three specific tyrosines: Tyr421, Tyr466 and Tyr482 for ensuring actin cross-linking modulation, cell migration improvement, invadopodia formation and interaction with proteins related to focal adhesion assembly [51, 61]. Interestingly, the levels of Tyr421 and Tyr466 (corresponding to Tyr421 and Tyr470 in human cortactin, respectively) were higher in metastatic sarcomatoid renal cell carcinoma (SRCC) patients compared to patients with non-metastatic carcinoma [62]; suggesting a direct role of this phosphorylation in tumour invasiveness. One of the pathways implicated in cortactin phosphorylation is PI3K/p-Akt activated by CXCR1/CXCL8 interaction [39].

In agreement with the hypothesis that an autocrine activation of CXCL8 receptors may be involved in maintaining an invasive phenotype in GB cells, treatment with the CXCR1/CXCR2 allosteric inhibitor in our experimental conditions, in which cells were not stimulated with exogenous CXCL8 (except for chemotaxis assay), resulted in a significant decrease of the ratio active MMP2/latent MMP2 paralleled with a marked decrease of the FAK(Tyr397) phosphorylation.

To dissect the specific pathways activated by the autocrine CXCL8 signalling in GB cells, we put to the evaluate the hypothesis that CXCR1/CXCR2 activation via PI3K may induce Akt phosphorylation at threonine 308 (Thr308) [63], thus triggering several signal cascades, including cortactin phosphorylation and also NF-κB activation [26, 29, 64, 65]. Treatment of U-87MG and GB primary cell culture with the allosteric inhibitor resulted in PI3K down regulation and a marked decrease of the p-Akt (Thr308) and p-cortactin (Tyr466) protein levels. DF2755A was also found to reduce NF-κB p65 nuclear protein levels with a parallel increase of IκBβ protein levels.

In addition to actin, also α-tubulin is essential for cytoskeletal dynamics responsible for cell invasiveness. Post-translational modifications occurring on α-tubulin are essential for microtubule and actin interactions that provide the basis for cell motility. α-tubulin acetylation occurs only at lysine 40 (Lys40) and plays a key role in intracellular trafficking, mitochondria-endoplasmic reticulum interactions and microtubule dynamics, such as their stabilization. Noteworthy during cell invasion, focal adhesion proteins regulate α-tubulin acetylation, which in turn ensures focal adhesions renewal [66]. Recently, in breast cancer cell lines and in breast cancer tissue levels of α-tubulin acetylation at Lys40 were directly correlated with highly invasive tumour phenotype [67]. Interestingly, in a study in which human liver protein-protein interactions were evaluated by yeast two-hybrid technology, an α-tubulin interaction with CXCR1 was identified [68].

In coherence with the proposed role of CXCR1/CXCR2 in the process of α-tubulin rearrangement, we observed an interesting reduction of acetylated α-tubulin (Lys40) levels in both treated GB cellular models compared to non-treated cellular models.

The cytoskeletal reorganization of actin microfilaments also regulates members of the Rho family of GTPases, such as Rho, Rac and Cdc42. The migrating cell needs anchor points on the ECM to move around the extracellular environment. Rac is required to regulate lamellipodia formation, while Cdc42 plays a central role in filopodia establishment. RhoA is required for formation and maintenance of focal adhesions and for cell tail retraction [69, 70], but RhoA takes part in further cellular functions, such as survival, motility, apoptosis and invasion [38, 39, 71].

In this context, it appears interesting the DF2755A-dependent down regulation of RhoA and Cdc42 protein levels, which results also in a down-regulation of p-FAK protein levels.

The reported results overall confirm that CXCR1/CXCR2 pathway is constitutively activated in GB cells and strongly influences cytoskeletal dynamics favouring cellular migration and invasiveness. This result on cell migration is in agreement with previous observations [20], even if in our experimental conditions DF2755A treatment did not affect cell proliferation.

Overall the reported data support the concept that CXCL8 production in unstimulated GB cells, possibly linked to the aberrant activation of NF-κB [26, 30], results in a constitutive activation of the cognate receptors and the activation of key pathways involved in cytoskeletal rearrangement and cell mobilization, which may account for the invasive potential of these cancer cells.

The observed reduction of α-tubulin and p-cortactin activation following DF2755A exposure is in line with our previous studies on the role of CXCR1 and CXCR2 on sensory neurons and confirm a link between CXCL8 signalling and cytoskeletal rearrangement in different cellular contexts [72].

Lastly, the inhibitory effect of CXCL8-CXCR1/CXCR2 axis on the Hippo pathway was investigated to further unravel the role of CXCL8 in conditioning the GB phenotype. The Hippo pathway is central to the regulation of organ size and tissue homeostasis. YAP and TAZ, two closely related transcription co-activators, are the downstream effectors of the Hippo pathway. Transcriptional inactivation of YAP and TAZ is due to phosphorylation mediated by Hippo kinases, such as LATS1/2. YAP and TAZ show the ability to translocate into the nucleus to ensure transcription of target genes. LATS1/2-dependent phosphorylation of YAP/TAZ allows their cytoplasmic sequestration and degradation by the proteasome. The Hippo pathway is affected by several extracellular and intracellular signals, such as cell-cell interaction, microenvironment ligands and metabolic by-products. Under specific circumstances, stable activation of YAP/TAZ is a typical feature of malignant human tumours [73]. It has been recently reported that attenuation of the Hippo signalling, resulting in upregulation of YAP/TAZ-mediated gene transcription, provides aggressive molecular features in glioblastoma multiforme [74, 75]. Preliminary results suggest a link between CXCR1/CXCR2 activation and the regulation of the Hippo/YAP/TAZ pathway. The Giα subunit of the heterotrimeric G-protein associated with CXCL8-CXCR1/CXCR2 signalling can inhibit LATS1/2 [76].

Further evidence on the connection between CXCL8 signalling pathway and YAP/TAZ was found in breast cancer cell lines, where YAP inhibition was associated with a low amount of CXCL8 secretion [77]. In our experimental conditions, treatment with the CXCR1/CXCR2 allosteric inhibitor resulted in induction of YAP and TAZ extra-nuclear localization, bearing in mind that these factors are typically constitutively present in the nucleus of GB cells. This is consistent with the role of the CXCL8-CXCR1/CXCR2 axis in inhibiting LATS1/2 responsible for YAP/TAZ phosphorylation and their subsequent inactivation.

**Figure 9:**
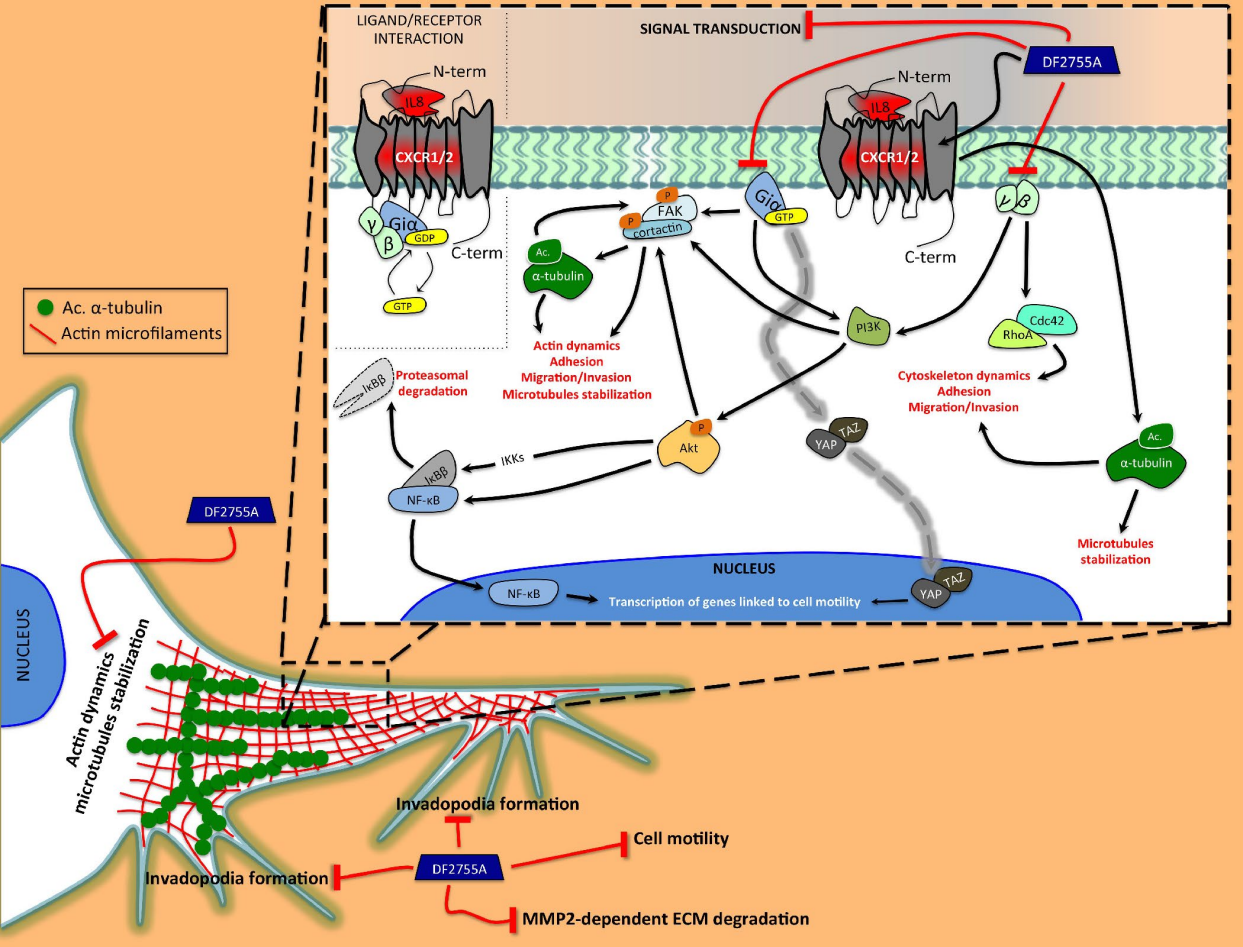
**Schematic representation of the proposed action mechanism of DF2755A in modulating the cytoskeletal dynamics.** Our study shows the central role of autocrine/paracrine CXCL8 and CXCR1/CXCR2 in activating the cellular mechanisms related to cell motility and cytoskeleton dynamics underlying GB invasiveness. The CXCL8 interaction with CXCR1/CXCR2 triggers the subunits of the heterotrimeric G-protein. The Giα subunit is able to directly induce the Fak phosphorylation at tyrosine 397, which in turn allows Fak-cortactin interaction. This interaction plays a key role in the regulation of cell motility and it is promoted by α-tubulin acetylation. Moreover, Giα can indirectly induce Fak phosphorylation by PI3K/Akt as well as could be involved in YAP/TAZ nuclear translocation. Instead, γ and β subunits can activate RhoA and Cdc42, proteins involved in cytoskeleton rearrangement occurring during cell migration. All GPCR subunits are positively linked to nuclear translocation of NF-κB p65 and IκBβ degradation. Interestingly, CXCR1/CXCR2 could, in some way, to be involved in α-tubulin acetylation on lysine 40, but this hypothetical correlation needs further investigation. DF2755A allosteric inhibitor shows the ability to adversely affect these CXCL8-CXCR1/CXCR2-dependent mechanisms resulting in a reduction of cellular motility. Black lines with arrowheads represent CXCL8-CXCR1/CXCR2-activated signalling pathways. Dashed gray line represents direct activation of YAP/TAZ nuclear translocation mediated by Giα subunit of CXCR1/CXCR2. Red lines represent inhibitory effects of DF2755A.

Overall, all the experiments performed support the concept that autocrine CXCL8-CXCR1/CXCR2 signalling plays a key role in the activation of an aggressive phenotype in glioblastoma cells, this effect is conserved both in U-87MG cell lines and in primary culture isolated from GB patient specimen. The significant effects of the allosteric inhibitor DF2755A in counteract the key pathways involved in migration and invasion of GB, suggest the potential of a pharmacological approach targeting CXCR1/CXCR2 pathways (summarized in Figure 9) to decrease migration and invasion of GB cells in the brain parenchyma, one of the principal mechanisms of recurrence.

## MATERIALS AND METHODS

### Materials

Dulbecco’s Modified Eagle’s Medium (DMEM), Foetal Bovine Serum (FBS), penicillin/streptomycin, glutamine, PBS and Trypsin-EDTA solution were purchased from Corning (Manassas, VA, USA). Hank’s Balance Salt Solution (HBSS), HEPES, Formaldehyde, Triton X-100, Bovine Serum Albumin (BSA), poly-L-lysine, DAPI, Trypan blue, Nonidet-P40, sodium deoxycholate, Sodium Dodecyl Sulphate (SDS), Tween 20, Igepal CA 630, Ethylenediamine tetra acetic acid (EDTA), phosphatase inhibitor cocktail 2, protease inhibitor cocktail, acrylamide/bis-acrylamide, Tris-(hydroxymethyl)-aminomethane (Tris), hydrogen chloride (HCl), sodium chloride (NaCl), Gelatin powder, Phalloidin-tetramethylrhodamine isothiocyanate (Phalloidin-TRITC) were all purchased from Sigma (St. Louis, Mo, USA). Subcellular Protein Fractionation Kit for Cultured Cells, Human IL-8 Platinum ELISA Kit, PVDF membranes, Micro BCA protein detection, Super Signal West Pico PLUS Chemiluminescent substrate were from Thermo Scientific (Rockford, IL, USA). Primary antibodies anti-p-FAK, anti-p-Akt, anti-YAP/TAZ, anti-Acetylated-α-Tubulin (Lys40) and anti-β-Actin-HRP conjugate were purchased from Cell Signaling Technology (Danvers, MA, USA). Anti-NF-κB p65, anti p-cortactin (Y466), anti-cortactin, anti-α-Tubulin and anti-PI3K p110 were purchased from Abcam (Cambridge, UK). Blotto non-fat dry milk, anti-RhoA, anti-Cdc42 and anti-Lamin B1 were from Santa Cruz Biotechnology (Dallas, TX, USA). Anti-FAK and anti-Akt were purchased from Sigma (St. Louis, Mo, USA), anti-IκB-β was from Thermo Scientific. Anti-p-Akt (Thr308) was purchased from Immunological Sciences (Rome, Italy). Anti-CXCR1 and anti-CXCR2 were from R&D Biosystem (Minneapolis, MI, USA). Western blotting secondary HRP-conjugated anti-mouse and anti-rabbit IgG antibodies were purchased from KLM bioscientific (San Diego, CA, USA). Secondary Alexa Fluor 488 and 633 conjugated anti-mouse and anti-rabbit IgG antibodies were purchased from Thermo Scientific. CellTiter 96 Aqueous One Solution Cell proliferation Assay was purchased from Promega (Madison, WI, USA). Vectashield mounting medium with DAPI from Vector Laboratories (Burlingame, CA, USA).

### Cell cultures

U-87MG (ATCC HTB-14) glioblastoma cell line was grown in Dulbecco’s Modified Eagle’s Medium (DMEM) supplemented with 10% heat inactivated foetal bovine serum (FBS), 1% of penicillin/streptomycin and 2 mM L-glutamine. The cell culture was incubated at 37°C in a 5% CO_2_ humidified atmosphere, the culture medium was replaced every two days. The subculture was obtained by using trypsin (0.05%)/ EDTA (0.53 mM), for 5 minutes at 37°C. An equal volume of culture medium was added to the cell suspension to inhibit trypsin digestion activity. Then the cells were centrifuged at 300 x *g* for 10 minutes. The cell pellet was suspended in culture medium and transferred to a new flask. All experiments were performed seeding the cells on day zero at 1 x 10^4^ cells/cm^2^. The next day the cells were treated with DF2755A for 24 hours at the indicated final concentrations.

### Glioblastoma primary cell culture

This study was ethically approved (Hospital Ethics Committee, n. 3729), and all patients were voluntary signing informed consent. Newly diagnosed GB patients (41 years to 70 years old, mean age of 60 years) were surgical resected at the Department of Neurosurgery, San Salvatore Hospital, L’Aquila, Italy. Individual tumour biopsies excluding necrotic fragments were maintained in culture medium and addressed to our laboratory. GB primary cell cultures were established as previously described [78]. The fragment was rinsed with Hank’s balanced salt solution (HBSS), the necrotic regions and red endothelial portions were removed. The obtaining fragments were finely minced into 0.5 mm^3^ section pieces approximately. Supernatant was discarded after cell suspension centrifugation at 260 x *g* for 5 minutes (for two time). Subsequently, by incubating the pellet with 4 mL of 0.125 % trypsin and 0.125% EDTA at 37°C for 10 minutes, the enzymatic digestion was obtained. Trypsin digestion activity was neutralized by adding growth culture medium containing 20% FBS. After centrifugation (5 minutes, 260 x *g*), the pellets were suspended in the culture growth medium, and then were transferred to 75 cm² flasks and maintained at 37 °C in 5% CO_2_, humidified atmosphere. The cells were cultured in DMEM supplemented with 20% FBS, 2 mM L-glutamine and 1% of penicillin/streptomycin and the medium was substituted thrice for a week. When the cell cultures reached the confluence they were divided in subcultures (1:2 or 1:3) by using trypsin (0.05%)/ EDTA (0.53 mM), for 5 minutes at 37 °C. The trypsin digestion activity was inhibited by adding an equal volume of culture medium and cells were centrifuged at 300 x *g* for 10 minutes. The obtained cell pellet was suspended in culture medium and transferred to new a flask. DMEM supplemented with 10% FBS, 2 mM L-glutamine and 1% of penicillin/streptomycin was used to maintain subculture cells and every three days the complete medium was changed. All experiments were carried out seeding the cells on day zero at 1 x 10^4^ cells/cm^2^. The next day the cells were treated with DF2755A for 24 hours at the indicated final concentrations. Experiments were performed considering three different primary cultures obtained from different patients. For each cell culture the experiments were performed in triplicated and data were pooled and submitted to statistical analysis.

### Treatments

DF2755A sodium salt monohydrate was dissolved in 1 ml of Phosphate Buffer pH 8.0-8.2 to obtain a 25 mM stock solution. DF2755A stock solution (25 mM) was freshly prepared for each experiment. All experiments were carried out seeding the cells on day zero at 1 x 10^4^ cells/cm^2^. 24 hours later the cells were treated with DF2755A for 24 hours. The stock solution was diluted in DMEM 10% FBS to obtain the indicated final concentration.

### Cell viability assay

MTS assay was used to evaluate the effect on cell viability upon 24 hours of exposition to DF2755A. Cell viability assay was performed as previously described [79].

### Detection of secreted CXCL8

To quantify the levels of secreted CXCL8 from the *in vitro* models, an enzymatic immunosorbent assay was used according to manufacturer’s instructions (Thermo Scientific, Rockford, IL, USA). The assay was performed on the supernatant of the collected complete culture medium from both cellular models (U-87MG and GB primary cell culture). The seeding density for collecting the supernatants was 1 x 10^4^ cells/cm^2^. The absorbance (450 nm) of the samples was measured with a spectrophotometric microplate reader (Perkin Elmer Victor3, Waltham, MA, USA). The amount of secreted CXCL8 was measured by extrapolation from a standard curve using CXCL8 as standard (15.6-1000 pg/ml). The results were reported as [pg/ml] of secreted CXCL8 value normalized on the corresponding cell number of the sample.

### Cytofluorimetric analysis

U-87MG cell line and GB primary cell culture were examined by FACS Calibur flow cytometry (BD Instruments Inc., Franklin Lakes, NJ, USA) for detection of the following markers: CXCR1 and CXCR2 both labelled with secondary antibodies Alexa Fluor 488 conjugate. The single cell suspension (2 x 10^6^ cells/ml) were washed with cold PBS and fixed, for 15 minutes at RT, with 2% formaldehyde in PBS. After each wash with cold PBS the cells were centrifuged at 300 x *g* for 5 minutes. For intracellular or total CXCR1/CXCR2 (indicated as tCXCR1 and tCXCR2) samples were permeabilized with 0.1% Triton X-100 in cold PBS for 5 minutes at RT. For surface CXCR1/2 (indicated as sCXCR1 and sCXCR2) samples were not permeabilized. Unspecific sites were blocked by 10% BSA in cold PBS for 20 minutes at RT. Subsequently, the cells were incubated with selected primary antibodies, anti-CXCR1 and anti-CXCR2 (both 1:100 and anti-mouse, diluted in 10% BSA in cold PBS) for 1 hour at RT. After washing with cold PBS, the cells were incubated for 45 minutes at RT with the secondary Alexa Fluor 488 conjugated anti-mouse IgG antibodies diluted in 10% BSA in cold PBS. Then the cells were washed with cold PBS and centrifuged 300 x *g* for 5 minutes. The pellet was suspended in 1 ml of cold PBS to perform the analysis. The cellular population of interest was gated according to its Forward Scatter (FSC)/Side Scatter (SSC) criteria. 10000 events were acquired for each sample and analysed by CellQuest software (BD Instruments Inc., Franklin Lakes, NJ, USA).

### Subcellular protein fractionation

To analyse NF-κB and YAP/TAZ nuclear protein levels, and to separate cytoplasmic proteins from nuclear proteins, the Subcellular Protein Fractionation Kit for Cultured Cells from Thermo Scientific was used according to manufacturer’s instructions. 24 hours before treatment the cells were seeded in T300 cm^2^ flask (seeding density 1 x 10^4^ cells/cm^2^). At the end of the treatment, the cells were harvested with trypsin-EDTA and centrifuged at 300 x *g* for 10 minutes. Then the cell pellet was washed with cold PBS and the cell suspension was transferred to a pre-chilled 1.5 ml microcentrifuge tube and centrifuged at 500 x *g* for 5 minutes. Cytoplasmic Extraction Buffer (CEB) was added to the cell pellet (200 μl for 2 x 10^6^ cells), and incubation at 4° C for 10 minutes with gentle mixing was performed. After centrifugation at 4° C for 5 minutes at 700 x *g* the cytoplasmic extract was collected to a pre-chilled tube and stored at -20° C. The remaining pellet was suspended in Membrane Extraction Buffer (MEB) (200 μl for 2 x 10^6^ cells) and the tube was vortexed for 10 seconds on the highest setting. Then the tube was incubated at 4° C for 10 minutes with gentle mixing. Later the tube was centrifuged at 4° C for 5 minutes at 3000 x *g* and the membrane extract was collected to a pre-chilled tube and stored at -20° C. Finally, Nuclear Extraction Buffer (NEB) was added to the pellet (100 μl for 2 x 10^6^ cells), the tube was vortexed for 20 seconds on the highest setting and incubated at 4° C for 30 minutes also in this case with gentle mixing. After centrifugation at 4° C for 5 minutes at 5000 x *g* the supernatant containing nuclear extract was transferred to a pre-chilled tube and stored at -20° C.

### Protein assay

The bicinchoninic acid method (Micro BCA protein detection, Thermo Scientific) was used to assess the total protein content. The colorimetric reaction was quantified by absorbance at 550 nm using a spectrophotometric microplate reader (Infinite F200 Tecan, Männedorf, Switzerland). By extrapolation from a BSA standard curve (0.025-2 mg/ml) the total amount of protein was quantified.

### Immunofluorescence analysis

U-87MG cell line and GB primary cell culture were allowed to adhere on poly-L-lysine coated glass coverslips (15 μg/ml) for 24 hours and then exposed to DF2755A for additional 24 hours. After the treatment, the cells were washed with PBS and immediately fixed with 4% paraformaldehyde in PBS, for 10 minutes at RT. The cells were washed thrice with PBS and then permeabilized with 0.1% Triton-X100 in PBS for 5 minutes at RT. The unspecific sites were blocked by incubation with 4% BSA in PBS for 20 minutes at RT. The cells were incubated overnight at 4°C with following primary antibodies: anti-RhoA 1:200, anti-YAP/TAZ 1:50 and with Phalloidin-TRITC (50 μg/ml; Sigma-Aldrich) all diluted in 4% BSA in PBS. Primary antibodies were revealed by secondary Alexa Fluor 488 (for anti-RhoA) or Alexa Fluor 633 (for anti-YAP/TAZ) conjugated anti-mouse and anti-rabbit IgG antibodies (incubation of 1 hour at RT), except for Phalloidin staining. After five washes with PBS, coverslips were mounted with Vectashield mounting medium with DAPI (Vector Laboratories Burlingame, CA, USA) and then observed at confocal laser microscope (Leica TCS SP5).

### Nuclear immunofluorescence quantification

For quantitative evaluation of cellular YAP/TAZ nuclear immunofluorescent signals, the cells were observed and photographed by confocal laser microscopy. Digital images (4 fields/condition, three replicates; range of cells analysed: 117-142 for GB primary cell culture, 167-181 for U-87MG) were analysed by ImageJ software (National Institutes of Health, Bethesda, MD) according to image processing package [80], as recommended by the manufacturer. To provide the signal intensity (in arbitrary units), the mean gray value was used.

### Western blotting analysis

Lysates from control and treated cells (30-60 μg of total proteins per sample) were run on 8-13% polyacrylamide SDS denaturing gels or 4-20% gradient polyacrylamide Mini-PROTEAN TGX Precast Gels (purchased from BIO-RAD, CA, USA). Running buffer 25 mM Tris and 192 mM Glycine was used for SDS-PAGE. The gels were run for 30 minutes at a constant voltage of 50 Volts, and about 2 hours at a constant voltage of 100 Volts. Proteins were transferred onto polyvinylidene difluoride (PVDF) sheets in blotting buffer 25 mM Tris, 192 mM Glycine and 20% Methanol by wet (constant 400 mA for 1 hour and 30 minutes) or semi-dry (constant 25 Volts 1 A for 1 hour and 30 minutes) electrophoretic transfer. Non-specific binding sites were blocked with blocking solution: 5% (w/v) non-fat dry milk in Tris-buffer saline containing 0.1% (v/v) Tween-20 (TBS-T). Membranes were incubated overnight at 4°C with the following primary antibodies diluted in blocking solution: anti-p-FAK (Tyr397) 1:500, anti-FAK 1:500, anti-p-Akt (Thr308) 1:500, anti-Akt 1:500, anti-p-Cortactin (Tyr466) 1:1000, anti-Cortactin 1:3000, anti-RhoA 1:500, anti-Cdc42 1:500, anti-Acetylated α-tubulin (Lys40) 1:1000, anti-α-tubulin 1:1000, anti-NF-κB p65 1:1000, anti-IκBβ 1:500, anti-PI3K p110 1:500, anti-YAP/TAZ 1:500, anti-Lamin B1 1:500 and anti-β-Actin HRP-conjugate 1:10000. Then, the membranes were washed thrice with TBS-T and then incubated, 1 hour at RT with gentle agitation, with secondary HRP-conjugated anti-mouse and anti-rabbit IgG antibodies diluted 1:10000 in blocking solution. After that, the membranes were washed four times with TBS-T. Immunoreactive bands were visualized by ECL, according to the manufacturer’s instructions. Bands from whole cell lysate obtained using Alliance 4.7 UVITEC (Cambridge, UK) were analysed by ImageJ software and normalized to β-actin and values were given as relative units (R.U.). All bands obtained from cytoplasmic and nuclear extract were analysed separately by ImageJ. For YAP/TAZ immunoblotting we used an antibody that recognizes both YAP and TAZ proteins. For this reason in the YAP/TAZ immunoblotting the cytoplasmic extract and nuclear extract were run separately since the nuclear extract required longer exposure time than cytoplasmic extract to observe the band of interest. In the cytoplasmic extract, high exposure time provided the saturation of the chemiluminescent signal. The bands obtained from cytoplasmic and nuclear extracts were analysed by ImageJ software and normalized to β-actin and Lamin B1, respectively, and values were given as R.U.

### Cell chemotaxis assay

Cell chemotaxis assay was carried out with the RTCA DP Instrument (ACEA Biosciences, San Diego, CA, USA) as recommended by the manufacturer's protocol and as already described [79]. Briefly, the core of the system is the microelectronic cell sensor arrays that are integrated into the bottom of an upper chamber, in close contact with a microporous membrane. Measuring the electronic impedance of these sensor electrodes allows to detect the changes in cells on the electrodes. 16-well plates called CIM-Plate have an upper chamber and a bottom chamber, separated by a microporous (8 μm of diameter) PET membrane that is coated with gelatin 0.1% [w/v]. Human CXCL8 (Miltenyi Biotec, Bergisch Gladbach, Germany) at the concentration of 20 ng/ml was used as a chemotactic agent in the bottom chamber. After 24 hours of treatment, the cells were suspended in FBS-free culture medium and placed in the upper chamber (1 x 10^5^ cells/well). Cell migration was monitored for 12 hours and represented using Normalized Cell Index (NCI) and discussed considering also the Slope parameter. NCI is used to measure the relative change in electrical impedance to represent cell status. CI calculation is based on the CI=(Z_i_-Z_0_)/15ς, where Z_i_ is the impedance at an individual point of time during the experiment, and Z_0_ is the impedance at the start of the experiment. Thus the NCI is a self-calibrated value derived from the ratio of measured impedances. Instead, the Slope (1/hr) measures how CI changes over time and can be used to determine the rates of these events. In our case, the Slope is indicated as a ratio between the value of DF2755A treated cells *vs* value of non-treated cells.

### Gelatin zymography

The culture supernatants from CIM-Plate upper chamber were collected after the chemotaxis assay and centrifuged at 400 x *g* for 5 minutes at 4°C. The supernatants were recovered and each 75 μl of clarified supernatant was mixed with 25 μl of sample buffer 4X (without beta mercaptoethanol), these samples were maintained for 10-15 minutes at RT. The loaded samples (30-60 μl for each lane) were subjected to 8% polyacrylamide gel electrophoresis containing 1 mg/ml gelatin powder (dissolved in the sterile water and incubated at 60° C for 20 min in a water bath). After electrophoresis, the gels were incubated with 2.5% Triton X-100 for 30 minutes at RT with gentle agitation. After that, the gels were washed three times with ddH_2_O and incubated in developing buffer at RT for 30 minutes with gentle agitation. In the end, the gels were incubated for 16–24 hours at 37° C with developing buffer. Subsequently, the gels were stained with 0.5% [w/v] Coomassie Blue R-250 for 1 hour at RT with gentle agitation and then gels were destained with a destaining solution (5% acetic acid, 10% methanol in ddH_2_O). Areas of digestion appear as clear bands against a blue darkly stained background. The images were obtained using an image scanner (gels were scanned), and the clear bands were analysed by ImageJ software and expressed as active MMP2/latent MMP2 ratio.

### Wound healing assay

GB primary cell culture and U-87MG cells (cell seeding density 1 x 10^4^ cells/well) were plated into 96-Well ImageLock™ plate (Essen Bioscience), and after 48 hours (GB primary cell culture) and 24 hours (U-87MG) of incubation the cell monolayers reached the confluence. Then cell monolayers were scratched with a wound marker™ tool, which allows obtaining high assay and imaging reproducibility in the scratched area. Non adherent cells were removed with two PBS washes and the adherent cells were incubated with fresh medium containing 10% FBS and the DF2755A treatment. Each treatment was performed in thirty-two replicates for GB primary cell culture and thirty-one replicates for U-87MG cells. The Incucyte™ Scratch Wound Software was used to acquire and analyse images from wound closure for 24 hours (2 images per well and per hour). This software utilizes three separate metrics to quantify cell migration over time. In this regard wound shrinkage is automatically monitored by Wound Width (μm). Changes in Wound Width are linked to cell migration.

### Statistical analysis

For primary cell culture data are mean of ± SEM of data obtained from three different primary cell cultures, each one assayed three times. Data were averaged out and statistical analysis were performed. For statistical analysis samples were processed by Graph Pad prism 6 software (RRID: SCR_002798). Statistical analysis was performed by the unpaired Student's t-test (with Welch’s correction). All experiments were performed a minimum of three biological replicates (n=3). *, p < 0.05; **, p < 0.01; ***, p < 0.001 were considered statistically significant. Data were expressed as mean ± SEM of three separate experiments (n=3).

## Supplementary Material

Supplementary Materials

Supplementary Figures
