# Supplementary material for: Autocrine CXCL8-dependent invasiveness triggers modulation of actin cytoskeletal network and cell dynamics
Source: Aging (Albany NY). 2020 Jan 27;12(2):1928–51. doi: 10.18632/aging.102733 (PMC7053615; doi:10.18632/aging.102733)
Supplement: Supplementary Materials [file aging-12-102733-s001..pdf]

SUPPLEMENTARY MATERIALS

Selectivity

DF2755A was tested at Eurofins Cerep SA (France) by radioligand binding assays to assess the off-target activities towards a panel of GPCRs, enzymes, ion channels, transporters and nuclear receptors. DF2755A was dissolved in DMSO to achieve 10 mM stock solution, which was diluted with water/HBSS to a final concentration of 10 μM. Cell membrane homogenates (48 μg protein) were incubated for 60 min at 22°C with the respective reference compound in the absence or presence of the test compound in a buffer containing 50 mM Tris-HCl (pH 7.4), 2 mM MgCl2 and 1 mM EDTA. After incubation, the samples were filtered rapidly under vacuum through glass fiber filters (GF/B, Packard Instruments, Meriden, CT, USA) presoaked with 0.3% polyethylenimine (PEI) and rinsed several times with ice-cold 50 mM Tris-HCl using a 96-sample cell harvester (Unifilter, Packard Instruments). The filters were dried, then counted for radioactivity in a scintillation counter (Topcount, Packard Instruments) using a scintillation cocktail (Microscint-O, Packard Instruments). The results were expressed as the percentage inhibition of the control radioligand-specific binding. The compounds were tested at a single concentration of 10 µM in triplicate.

Tested targets were as follows: GPCR: A2A (agonist radioligand), α1A (antagonist radioligand), α2A (antagonist radioligand), β1 (agonist radioligand), β2 (agonist radioligand), BK1 (antagonist and agonist radioligand), BK2 (antagonist and agonist radioligand), CB1 (antagonist and agonist radioligand), CB2 (antagonist and agonist radioligand), CCK1 (CCKA) (agonist radioligand), D1 (antagonist radioligand), D2 (antagonist and agonist radioligand), D3 (antagonist and agonist radioligand), ETA (agonist radioligand), H1(antagonist radioligand), H2 (antagonist radioligand), M1 (antagonist radioligand), M2 (antagonist and agonist radioligand), M3 (antagonist radioligand), NK1(agonist radioligand), δ(DOP) (agonist radioligand), κ(KOP) (agonist radioligand), μ(MOP) (agonist radioligand), ORL1 (agonist radioligand), 5-HT1A (agonist radioligand), 5-HT1B (antagonist radioligand), 5-HT2A (agonist radioligand), 5-HT2B (agonist radioligand), V1a (agonist radioligand). Transporters: 5-HT transporter (antagonist radioligand), dopamine transporter (antagonist radioligand), norepinephrine transporter (antagonist radioligand). Ion Channels: 5-HT3 (antagonist radioligand), BZD (central) (agonist radioligand), NMDA (antagonist radioligand), N neuronal alpha4beta2 (agonist radioligand), Ca2+channel (L.dihydropyridine site) (antagonist radioligand), Na+channel (site 2) (antagonist radioligand), KV (antagonist radioligand). Nuclear Receptors: AR (agonist radioligand), GR (agonist radioligand). Kinases and other non-kinase enzymes: CTK Lck kinase, acetylcholinesterase, PDE3A, PDE4D2, MAO-A (antagonist radioligand).

Finally, DF2755A was tested on TRPM8, TRPV1, TRPV4, TRPA1 and Nav1.7 ion channels in agonist and antagonist mode. TRPM8-, TRPA1-, TRPV1-, TRPV4- and Nav1.7-expressing HEK-293 cells were analyzed in order to study the response to the compounds using a Ca2+ mobilization-dependent fluorescence signal in 384 MTP format. Cells were seeded at 10,000 cells per well in 384 MTP in complete medium (25 μl well-1). Twenty-four hours after seeding, the medium was removed and cells were loaded with 20 μL/well of the Fluo-8 NW dye solution. The dye-loaded cell plates were incubated for 1 h at RT. Test compounds at 3X-concentration in 1.5% DMSO Tyrode's buffer were added to the wells of an assay plate, in 10 μL volume (for a final DMSO concentration of 0.5%) and read by the FLIPRTETRA plate. The kinetic response was monitored by the instrument over a period of 3 mi (180 seconds). A second injection of 10 μL well-1 of reference agonists (Capsaicin, GSK1016790A, Isothiocyanate and Veratridine for TRPA1, TRPV1, TRPV4 and Nav1.7, respectevely) at 4X-concentration in assay buffer (EC80) was added by the FLIPRTETRA. The signal of the emitted fluorescence was recorded for an additional 3 min.

DF2755A was tested at 8 concentrations in quadruplicate (30 μM was the highest tested concentration) to determinate the IC50. The compound curve fitting profile on each dose-response was performed with the Condoseo module of Genedata Screener 13.0.5.

RESULTS

DF2755A activities towards a panel of GPCRs, enzymes, ion channels, transporters nuclear receptors and kinases revealed no inhibitory properties of the compound.
