# Supplementary material for: Autocrine CXCL8-dependent invasiveness triggers modulation of actin cytoskeletal network and cell dynamics
Source: Aging (Albany NY). 2020 Jan 27;12(2):1928–51. doi: 10.18632/aging.102733 (PMC7053615; doi:10.18632/aging.102733)
Supplement: Supplementary Figures [file aging-12-102733-s002..pdf]

SUPPLEMENTARY FIGURES

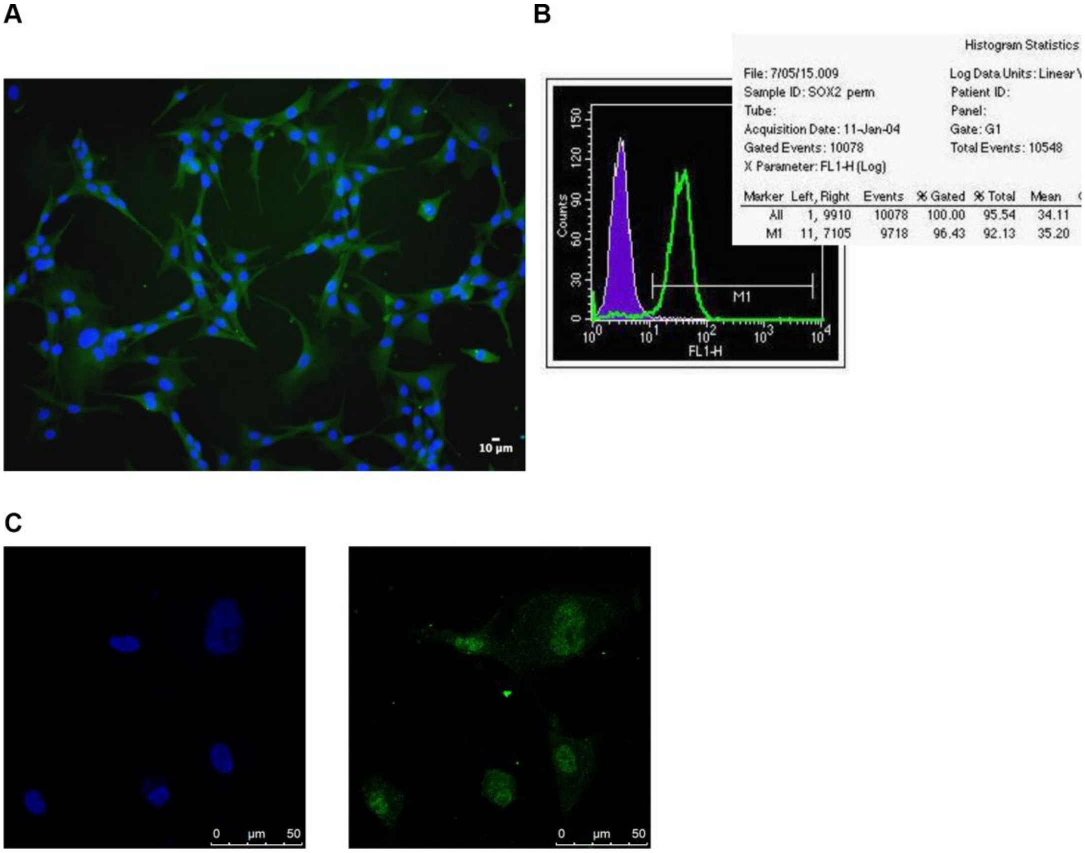

Supplementary Figure 1. Characterization analysis of the primary cell cultures, the data reported are representative of one primary cell culture derived from one patient specimen. (**A**) GFAP (glial fibrillary acid protein) immunostaining (green), (**B**) flow cytometry assay to quantify SOX2-positive primary cell population. The positive cells represent the 92% of the whole cell population. (**C**) SOX2 immunostaining (green) and DAPI staining of nuclei (blue) of primary cell culture. Bar = 10µm and 50µm.

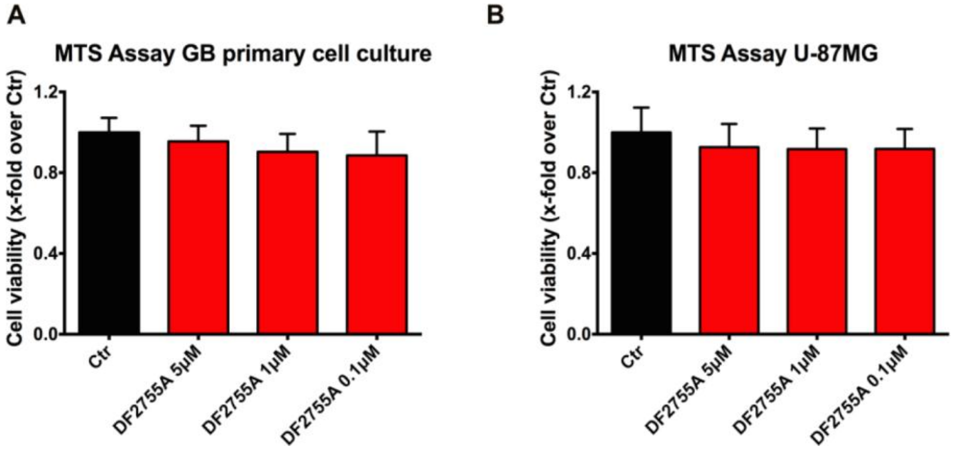

Supplementary Figure 2. DF2755A effect on GB cellular models viability. To evaluate DF2755A effect on cellular viability MTS assay was performed in GB primary cell culture (**A**) and U-87MG cell line (**B**). There is not significantly effect on cell viability as shown by the histograms. Data are means ± SEM of 3 different experiments performed in quadruplicate.
